# Biologically feasible gene trees, reconciliation maps and informative triples

**DOI:** 10.1186/s13015-017-0114-z

**Published:** 2017-08-29

**Authors:** Marc Hellmuth

**Affiliations:** 1 Institute of Mathematics and Computer Science, University of Greifswald, Walther-Rathenau-Strasse 47, 17487 Greifswald, Germany; 2 Center for Bioinformatics, Saarland University, Building E 2.1, P.O. Box 151150, 66041 Saarbrücken, Germany

**Keywords:** DTL-scenario, Reconciliation, Horizontal gene transfer, Phylogenetic tree, Triples, Event-label

## Abstract

**Background:**

The history of gene families—which are equivalent to *event-labeled* gene trees—can be reconstructed from empirically estimated evolutionary event-relations containing pairs of orthologous, paralogous or xenologous genes. The question then arises as whether inferred event-labeled gene trees are *biologically feasible*, that is, if there is a possible true history that would explain a given gene tree. In practice, this problem is boiled down to finding a reconciliation map—also known as DTL-scenario—between the event-labeled gene trees and a (possibly unknown) species tree.

**Results:**

In this contribution, we first characterize whether there is a valid reconciliation map for binary event-labeled gene trees *T* that contain speciation, duplication and horizontal gene transfer events and some unknown species tree *S* in terms of “informative” triples that are displayed in *T* and provide information of the topology of *S*. These informative triples are used to infer the unknown species tree *S* for *T*. We obtain a similar result for non-binary gene trees. To this end, however, the reconciliation map needs to be further restricted. We provide a polynomial-time algorithm to decide whether there is a species tree for a given event-labeled gene tree, and in the positive case, to construct the species tree and the respective (restricted) reconciliation map. However, informative triples as well as DTL-scenarios have their limitations when they are used to explain the biological feasibility of gene trees. While reconciliation maps imply biological feasibility, we show that the converse is not true in general. Moreover, we show that informative triples neither provide enough information to characterize “relaxed” DTL-scenarios nor non-restricted reconciliation maps for non-binary biologically feasible gene trees.

## Background

The evolutionary history of genes is intimately linked with the history of the species in which they reside. Genes are passed from generation to generation to the offspring. Some of those genes are frequently duplicated, mutate, or get lost—a mechanism that also ensures that new species can evolve. In particular, genes that share a common origin (*homologs*) can be classified into the type of their “evolutionary event relationship”, namely *orthologs*, *paralogs* and *xenologs* [1, 2]. Two homologous genes are *orthologous* if at their most recent point of origin the ancestral gene is transmitted to two daughter lineages; a *speciation* event happened. They are *paralogous* if the ancestor gene at their most recent point of origin was duplicated within a single ancestral genome; a *duplication* event happened. Horizontal gene transfer (HGT) refers to the transfer of genes between organisms in a manner other than traditional reproduction and across different species and yield so-called *xenologs*. In contrast to orthology and paralogy, the definition of xenology is less well established and by no means consistent in the biological literature. One definition stipulates that two genes are *xenologs* if their history since their common ancestor involves horizontal transfer of at least one of them [2, 3]. The mathematical framework for evolutionary event-relations relations in terms of symbolic ultrametrics, cographs and two-structures [4–7], on the other hand, naturally accommodates more than two types of events associated with the internal nodes of the gene tree. We follow the notion in [1, 6] and call two genes xenologous, whenever their least common ancestor was a HGT event.

The knowledge of evolutionary event relations such as orthology, paralogy or xenology is of fundamental importance in many fields of mathematical and computational biology, including the reconstruction of evolutionary relationships across species [8–12], as well as functional genomics and gene organization in species [13–15]. The type of event relationship is determined by the true history of the genes and species. However, events of the past cannot be observed directly and hence, must be inferred from the genomic data available today. Tree-reconciliation methods are widely studied in the literature [9, 16–31] and provide one way to address this problem. Here, a gene tree is mapped into a species tree such that certain optimization criteria are fulfilled. This mapping, eventually, identifies inner vertices of the gene tree as a duplication, speciation or HGT. These methods usually require a gene and species tree as input. In most practical applications, however, neither the gene tree nor the species tree can be determined unambiguously. Intriguingly, there are methods to infer orthologs [14, 32–40] or to detect HGT [41–45] *without* the need to construct gene or species trees. Given empirical estimated event-relations one can infer the history of gene families which are equivalent to event-labeled gene trees [5, 6, 11, 46–48].

The crucial point is the following important result: For (tree-free estimated) event-relations there is an event-labeled gene tree that represents this estimate if and only if the respective event-relations are directed cographs [5, 6]. Usually, estimated event-relations violate this condition and must, therefore, be corrected [33, 36, 46–51]. Such corrected event-relations can, in most cases, be represented by an event-labeled gene tree. However, these trees can still be error-prone in the sense that there is no species tree on which they can evolve. The latter strongly depends on the applied correction method, the presence or absence of HGT events and, in particular, the theoretical model that is used to define that “a gene tree evolves along a species tree” (reconciliation map). The method ParaPhylo [11] already uses many of the latter mentioned ideas for the reconstruction of species trees and event-labeled gene trees without HGT-events. ParaPhylo is based on the knowledge of estimated orthology relations which are cleaned up to the closest cograph and, afterwards, corrected to obtain biologically feasible gene trees.

For an event-labeled gene tree to be biologically feasible there must be a putative “true” history that can explain the inferred gene tree. However, in practice it is not possible to observe the entire evolutionary history as, e.g. gene losses eradicate the entire information on parts of the history. Therefore, the problem of determining whether an event-labeled gene tree is biologically feasible is reduced to the problem of finding a valid reconciliation map, also known as DTL-scenario [29, 31], between the event-labeled gene trees and an arbitrary (possibly unknown) species tree. DTL-scenarios and its variants have been extensively studied [22, 29, 52–54] and have also applications in the context of the host-parasite cophylogeny problem [55–62].

In this contribution, we assume that we have a given event-labeled gene tree *T* and wish to answer the question: *Is* *T* *biologically feasible and how much information about the* *unknown* *species tree* *S* *and the reconciliation between* *T* *and* *S* *is already contained in the gene tree* *T*?

To this end, we first provide a mathematical definition of the term “biologically feasible” and two types of reconciliation maps: DTL-scenarios (as used in, e.g. [29, 31, 63]) and a restricted version (as used in, e.g. [12, 48]). Given the event-labeled gene-trees, it is possible to derive “informative” triples that are displayed in the gene tree *T* and provide information on the topology of the species tree *S*. In particular, we prove that consistency of informative triple sets characterize whether there are DTL-scenarios and restricted maps for binary and non-binary gene trees, respectively. The latter generalizes results established for binary gene trees that do not contain HGT-events by Hernandez et al. [10]. Furthermore, we provide a polynomial-time algorithm to decide whether there is a species tree for a given event-labeled gene tree and, in the positive case, to construct the species tree and a respective (restricted) reconciliation map.

In addition to the established results, we discuss limitations of reconciliation maps to explain biological feasibility of gene trees. While any (restricted) reconciliation map gives an idea of a putative true history that can explain the given gene tree, the converse is in general not true. We provide simple examples that show that not all biologically feasible gene trees can be explained by (restricted) DTL-scenarios. This immediately raises the question whether generalization of reconciliation maps might be used to explain biological feasibility. We shortly discuss a mild generalization, so-called “relaxed” reconciliation maps. However, as it turns out such general maps cannot be characterized by informative triples. We close this contribution with a couple of open problems.

## Preliminaries

A *rooted tree* $T=(V,E)$ (*on* *L*) is an acyclic connected simple graph with leaf set $L\subseteq V$, set of edges *E*, and set of interior vertices $V^0=V\setminus L$ such that there is one distinguished vertex $\rho _T \in V$, called the *root of* *T*.

A vertex $v\in V$ is called a *descendant* of $u\in V$, $v \preceq _T u$, and *u* is an *ancestor* of *v*, $u \succeq _T v$, if *u* lies on the path from $\rho _T$ to *v*. As usual, we write $v \prec _T u$ and $u \succ _T v$ to mean $v \preceq _T u$ and $u\ne v$. If $u \preceq _T v$ or $v \preceq _T u$ then *u* and *v* are *comparable* and otherwise, *incomparable*. For $x\in V$, we write $L_T(x):=\{ y\in L \mid y\preceq x\}$ for the set of leaves in the subtree *T*(*x*) of *T* rooted in *x*.

### Remark 1

It will be convenient to use a notation for edges *e* that implies which of the vertex in *e* is closer to the root. Thus, the notation for edges (*u*, *v*) of a tree is always chosen such that $u\succ _T v$.

For our discussion below we need to extend the ancestor relation $\preceq _T$ on *V* to the union of the edge and vertex sets of *T*. More precisely, for the edge $e=(u,v)\in E$ we put $x \prec _T e$ if and only if $x\preceq _T v$ and $e \prec _T x$ if and only if $u\preceq _T x$. For edges $e=(u,v)$ and $f=(a,b)$ in *T* we put $e\preceq _T f$ if and only if $v \preceq _T b$. In the latter case, the edges *e* and *f* are called comparable.

For a non-empty subset of leaves $A\subseteq L$, we define ${\text {lca}}_T(A)$, or the *least common ancestor of* *A*, to be the unique $\preceq _T$-minimal vertex of *T* that is an ancestor of every vertex in *A*. In case $A=\{x,y \}$, we put ${\text {lca}}_T(x,y):={\text {lca}}_T(\{x,y\})$ and if $A=\{x,y,z \}$, we put ${\text {lca}}_T(x,y,z):={\text {lca}}_T(\{x,y,z\})$. We will make frequent use that for two non-empty vertex sets *A*, *B* of a tree, it always holds that ${\text {lca}}(A\cup B) = {\text {lca}}({\text {lca}}(A),{\text {lca}}(B))$.

A *phylogenetic tree* *T* (*on* *L*) is a rooted tree $T=(V,E)$ (on *L*) such that no interior vertex $v\in V^0$ has degree two, except possibly the root $\rho _T$. If *L* corresponds to a *set of genes* $\mathbb {G}$ or *species* $\mathbb {S}$, we call a phylogenetic tree on *L* *gene tree* and *species tree*, respectively. The *restriction* $T_{|L'}$ of a phylogenetic tree *T* to $L'\subseteq L$ is the rooted tree with leaf set $L'$ obtained from *T* by first forming the minimal subtree of *T* with leaf set $L'$ and then by suppressing all vertices of degree two with the exception of the root $\rho _{T_{|L'}}$. By construction, $V(T_{|L'})\subseteq V(T)$. If $T=(V,E)$ is equipped with a map $\ell :V\cup E \rightarrow M$, then the restriction of $\ell$ to $T_{|L'}=(V',E')$ is the map $\ell _{|L'}:V'\cup E' \rightarrow M$ that satisfies

$$\begin{aligned} \ell _{|L'}(\alpha ) =\left\{ \begin{array}{ll} \ell (v) &{}\quad \text{ if } \alpha = v\in V',\ \\ \ell (e) &{}\quad \text{ if } \alpha =(u,b)\in E', e=(u,v)\in E \\ &{}\quad \text {and either } v=b \text { or } v \text { is suppressed} \\ &{} \quad \text {in } T_{|L'} \text { and lies on the path from } \\ &{} \quad u \text { to } { b} \text { in } T.\ \end{array}\right. \end{aligned}$$

In other words, $\ell _{|L'}$ keeps the vertex-labels of all non-suppressed vertices and assigns the edge-label of the edge (*u*, *v*) in *T* to the edge (*u*, *v*) in $T_{|L'}$, if $v=b$ and otherwise, to the edge (*u*, *b*) in $T_{|L'}$, where *b* is the first non-suppressed vertex that lies on the unique path from *v* to *b* in *T*.

Rooted triples are phylogenetic trees on three leaves with precisely two interior vertices. They constitute an important concept in the context of supertree reconstruction [64–66] and will also play a major role here. A rooted tree *T* on *L* *displays* a triple $\mathsf {(xy|z)}$ if, $x,y,z\in L$ and the path from *x* to *y* does not intersect the path from *z* to the root $\rho _T$ and thus, having ${\text {lca}}_T(x,y)\prec _T {\text {lca}}_T(x,y,z)$. We denote by $\mathcal {R}(T)$ the set of all triples that are displayed by the rooted tree *T*.

A set *R* of triples is *consistent* if there is a rooted tree *T* on $L_R= \cup _{r\in R} L_r(\rho _r)$ such that $R\subseteq \mathcal {R}(T)$ and thus, *T* *displays* each triple in *R*. Not all sets of triples are consistent of course. Nevertheless, given a triple set *R* there is a polynomial-time algorithm, referred to in [64, 67] as BUILD, that either constructs a phylogenetic tree *T* that displays *R* or that recognizes that *R* is not consistent [68]. The runtime of BUILD is $\mathcal {O}(|L_R||R|)$ [64]. Further practical implementations and improvements have been discussed in [69–72].

We will consider rooted trees $T=(V,E)$ from which particular edges are removed. Let $\mathcal {E}\subseteq E$ and consider the forest $T_{\mathcal {\overline{E}}}:=(V,E\setminus \mathcal {E})$. We can preserve the order $\preceq _T$ for all vertices within one connected component of $T_{\mathcal {\overline{E}}}$ and define $\preceq _{T_{\mathcal {\overline{E}}}}$ as follows: $x\preceq _{T_{\mathcal {\overline{E}}}}y$ iff $x\preceq _{T}y$ and *x*, *y* are in same connected component of $T_{\mathcal {\overline{E}}}$. Since each connected component $T'$ of $T_{\mathcal {\overline{E}}}$ is a tree, the ordering $\preceq _{T_{\mathcal {\overline{E}}}}$ also implies a root $\rho _{T'}$ for each $T'$, that is, $x\preceq _{T_{\mathcal {\overline{E}}}} \rho _{T'}$ for all $x\in V(T')$. If $L(T_{\mathcal {\overline{E}}})$ is the leaf set of $T_{\mathcal {\overline{E}}}$, we define $L_{T_{\mathcal {\overline{E}}}}(x) = \{y\in L(T_{\mathcal {\overline{E}}}) \mid y\prec _{T_{\mathcal {\overline{E}}}} x\}$ as the set of leaves in $T_{\mathcal {\overline{E}}}$ that are reachable from *x*. Hence, all $y\in L_{T_{\mathcal {\overline{E}}}}(x)$ must be contained in the same connected component of $T_{\mathcal {\overline{E}}}$. We say that the forest $T_{\mathcal {\overline{E}}}$ displays a triple *r*, if *r* is displayed by one of its connected components. Moreover, $\mathcal {R}(T_{\mathcal {\overline{E}}})$ denotes the set of all triples that are displayed by the forest $T_{\mathcal {\overline{E}}}$.

## Biologically feasible and observable gene trees

A gene tree arises through a series of events (speciation, duplication, HGT, and gene loss) along a species tree. In a “true history” the gene tree ${\widehat{T}} = (V,E)$ on a set of genes ${\widehat{\mathbb {G}}}$ is equipped with an *event-labeling* map ${\widehat{t}}:V\cup E\rightarrow {\widehat{I}}\cup \{0,1\}$ with ${\widehat{I}}=\{\mathfrak {s},\mathfrak {d},\mathfrak {t},\odot ,\varvec{\mathsf {x}}\}$ that assigns to each vertex *v* of ${\widehat{T}}$ a value ${\widehat{t}}(v)\in {\widehat{I}}$ indicating whether *v* is a speciation event ($\mathfrak {s}$), duplication event ($\mathfrak {d}$), HGT event ($\mathfrak {t}$), extant leaf ($\odot$) or a loss event ($\varvec{\mathsf {x}}$). Note, in the figures we omitted the symbol $\odot$ and used $\bullet , \square$ and $\triangle$ for $\mathfrak {s}, \mathfrak {d}$ and $\mathfrak {t}$, respectively.

**Fig. 1:**
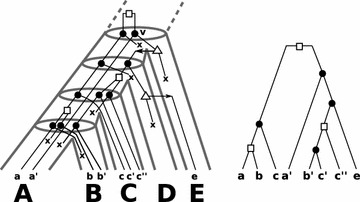
*Left* an example of a “true” history of a gene tree that evolves along the (*tube-like*) species tree. The set of extant genes $\mathbb {G}$ comprises* a*,*a*′,*b*,*b*′,*c*,*c*′,*c*″ and *e* and $\sigma$ maps each gene in $\mathbb {G}$ to the species (*capitals below * the genes) $A,B,C,E\in \sigma (\mathbb {G})$. For simplicity all speciation events followed by a loss along the path from *v* to $a'$ in *T* are omitted. *Left* the observable gene tree $(T;t,\sigma )$ is shown. Since there is a true scenario which explains $(T;t,\sigma )$, the gene tree is biologically feasible. In particular, $(T;t,\sigma )$ satisfies (O1), (O2) and (O3)

Horizontal gene transfer is intrinsically a directional event, i.e., there is a clear distinction between the horizontally transferred “copy” and the “original” that continues to be vertically transferred. To this end, the edges in the gene tree are annotated by associating a label to the edge that points from the horizontal transfer event to the next event in the history of the copy. To be more precise, to each edge *e* a value ${\widehat{t}}(e)\in \{0,1\}$ is assigned that indicates whether *e* is a *transfer edge* (1) or not (0). Hence, $e=(x,y)$ and ${\widehat{t}}(e) =1$ iff ${\widehat{t}}(x)=\mathfrak {t}$ and the genetic material is transferred from the species containing *x* to a species containing *y*. We remark that the restriction of *t* to the vertex set *V* was introduced as “symbolic dating map” in [4] and that there is a close relationship to so-called cographs [5, 73, 74]. Let $\mathbb {G}\subseteq {\widehat{\mathbb {G}}}$ be the set of all extant genes in ${\widehat{T}}$, i.e., $\mathbb {G}$ contains all genes *v* of ${\widehat{\mathbb {G}}}$ with ${\widehat{t}}(v)\ne \varvec{\mathsf {x}}$. Hence, there is a map $\sigma :\mathbb {G}\rightarrow \mathbb {S}$ that assigns to each extant gene the extant species in which it resides.

We assume that the gene tree and its event labels are inferred from (sequence) data, i.e., *T* is restricted to those labeled trees that can be constructed at least in principle from observable data. Gene losses eradicate the entire information on parts of the history and thus, cannot directly be observed from extant sequences. Hence, in our setting the (observable) gene tree *T* is the restriction ${\widehat{T}}_{|\mathbb {G}}$ to the set of extant genes equipped with the event-label $t={\widehat{t}}_{|\mathbb {G}}$, see Fig. 1. Since all leaves of *T* are extant genes in $\mathbb {G}$ we don’t need to specially label the leaves in $\mathbb {G}$, and thus simplify the event-labeling map $t:V^0\cup E\rightarrow I\cup \{0,1\}$ by assigning only to the interior vertex an event in $I=\{\mathfrak {s},\mathfrak {d},\mathfrak {t}\}$. We assume here that all non-transfer edges transmit the genetic material vertically, that is, from an ancestral species to its descendants.

### **Definition 1**

We write $(T;t,\sigma )$ for the tree $T=(V,E)$ with event-labeling *t* and corresponding map $\sigma$. The set $\mathcal {E}= \{e\in E\mid t(e)=1\}$ will always denote the set of transfer edges in $(T;t,\sigma )$.

Additionally, we consider gene trees $(T=(V,E);t,\sigma )$ from which the transfer edges have been removed, resulting in the forest $T_{\mathcal {\overline{E}}}= (V, E\setminus \mathcal {E})$ in which we preserve the event-labeling *t* of all vertices.

We call a gene tree $(T;t,\sigma )$ on $\mathbb {G}$ *biologically feasible*, if there is a true scenario such that $T = {\widehat{T}}_{|\mathbb {G}}$ and $t={\widehat{t}}_{|\mathbb {G}}$, that is, there is a true history that can explain $(T;t,\sigma )$. By way of example, the gene tree in Fig. 1 (right) is biologically feasibly. However, so-far it is unknown whether there are gene trees $(T;t,\sigma )$ that are not biologically feasible. Answering the latter might be a hard task, as many HGT or duplication vertices followed by losses can be inserted into *T* that may result in a putative true history that explains the event-labeled gene tree.

Following Nøjgaard et al. [63], we additionally restrict the set of observable gene trees $(T;t,\sigma )$ to those gene trees that satisfy the following observability axioms:

1. Every internal vertex *v* has degree at least three, except possibly the root which has degree at least two.
2. Every HGT node has at least one transfer edge, $t(e)=1$, and at least one non-transfer edge, $t(e)=0$.
3. (*a*) If $x\in V$ is a speciation vertex, then there are distinct children *v*, *w* of *x* in *T* with $\sigma _{T_{\mathcal {\overline{E}}}}(v)\cap \sigma _{T_{\mathcal {\overline{E}}}}(w) = \emptyset$. (*b*) If $(x,y) \in \mathcal {E}$, then $\sigma _{T_{\mathcal {\overline{E}}}}(x)\cap \sigma _{T_{\mathcal {\overline{E}}}}(y) = \emptyset$.

Condition (O1) is justified by the restriction $T={\widehat{T}}_{|\mathbb {G}}$ of the true binary gene tree ${\widehat{T}}$ to the set of extant genes $\mathbb {G}$, since $T={\widehat{T}}_{|\mathbb {G}}$ is always a *phylogenetic* tree. In particular, (O1) ensures that every event leaves a historical trace in the sense that there are at least two children that have survived in at least two of its subtrees. Condition (O2) ensures that for an HGT event a historical trace remains of both the transferred and the non-transferred copy.

Condition (O3.a) is a consequence of (O1), (O2) and a stronger Condition (O3.a’) claimed in [63]: *If* *x* *is a speciation vertex, then there are at least two distinct children* *v*, *w* of *x* *such that the species* *V* *and* *W* *that contain* *v* *and* *w*, *resp., are incomparable in* *S*. Note, a speciation vertex *x* cannot be observed from data if it does not “separate” lineages, that is, there are two leaf descendants of distinct children of *x* that are in distinct species. Condition (O3.a’) is even weaker and ensures that any “observable” speciation vertex *x* separates at least locally two lineages. As a result of (O3.a’) one can obtain (O3.a) [63]. Intuitively, (O3.a) is satisfied since within a connected component of $T_{\mathcal {\overline{E}}}$ no genetic material is exchanged between non-comparable nodes. Thus, a gene separated in a speciation event necessarily ends up in distinct species in the absence of the transfer edges.

Condition (O3.b) is a consequence of (O1), (O2) and a stronger Condition (O3.b’) claimed in [63]: *If* (*v*, *w*) *is a transfer edge in* *T*, *then* $t(v)=\mathfrak {t}$ *and the species* *V* *and* *W* *that contain* *v* *and* *w*, *resp., are incomparable in* *S*. Note, if $(v,w)\in \mathcal {E}$ then *v* signifies the transfer event itself but *w* refers to the next (visible) event in the gene tree *T*. In a “true history” *v* is contained in a species *V* that transmits its genetic material (maybe along a path of transfers) to a contemporary species *Z* that is an ancestor of the species *W* containing *w*. In order to have evidence that this transfer happened, Condition (O3.b’) is used and as a result one obtains (O3.b). The intuition behind (O3.b) is as follows: observe that ${T_{\mathcal {\overline{E}}}}(x)$ and ${T_{\mathcal {\overline{E}}}}(y)$ are subtrees of distinct connected components of $T_{\mathcal {\overline{E}}}$ whenever $(x,y) \in \mathcal {E}$. Since HGT amounts to the transfer of genetic material *across* distinct species, the genes *x* and *y* are in distinct species, cf. (O3.b). However, since $T_{\mathcal {\overline{E}}}$ does not contain transfer edges and thus, there is no genetic material transferred across distinct species *between* distinct connected components in $T_{\mathcal {\overline{E}}}$. We refer to [63] for further details.

### Remark 2

In what follows, we only consider gene trees $(T;t,\sigma )$ that satisfy (O1), (O2) and (O3).

We simplify the notation a bit and write $\sigma _{T_{\mathcal {\overline{E}}}}(u):=\sigma (L_{T_{\mathcal {\overline{E}}}}(u))$.

Based on Axiom (O2) the following results was established in [63].

### **Lemma 3.1**

Let $(T;t,\sigma )$ *be an event-labeled gene tree. Let* $\mathcal {T}_1, \dots , \mathcal {T}_k$ *be the connected components of* $T_{\mathcal {\overline{E}}}$ *with roots* $\rho _1, \dots , \rho _k$ *, respectively. Then,* $\{L_{T_{\mathcal {\overline{E}}}}(\rho _1), \dots , L_{T_{\mathcal {\overline{E}}}}(\rho _k)\}$ *forms a partition of* $\mathbb {G}$.

Lemma 3.1 particularly implies that $\sigma _{T_{\mathcal {\overline{E}}}}(x) \ne \emptyset$ for all $x\in V(T)$. Note, $T_{\mathcal {\overline{E}}}$ might contain interior vertices (distinct from the root) that have degree two. Nevertheless, for each $x\preceq _{T_{\mathcal {\overline{E}}}} y$ in $T_{\mathcal {\overline{E}}}$ we have $x\preceq _T y$ in *T*. Hence, partial information (that in particular is “undisturbed” by transfer edges) on the partial ordering of the vertices in *T* can be inferred from $T_{\mathcal {\overline{E}}}$.

## Reconciliation map

Before we define a reconciliation map that “embeds” a given gene tree into a given species tree we need a slight modification of the species tree. In order to account for duplication events that occurred before the first speciation event, we need to add an extra vertex and an extra edge “above” the last common ancestor of all species: hence, we add an additional vertex to *W* (that is now the new root $\rho _S$ of *S*) and the additional edge $(\rho _S,{\text {lca}}_S(\mathbb {S}))\in F$. Note that strictly speaking *S* is not a phylogenetic tree anymore. In case there is no danger of confusion, we will from now on refer to a phylogenetic tree on $\mathbb {S}$ with this extra edge and vertex added as a species tree on $\mathbb {S}$.

### **Definition 2**

(DTL-scenario) Suppose that $\mathbb {S}$ is a set of species, $S=(W,F)$ is a phylogenetic tree on $\mathbb {S}$, $T=(V,E)$ is a gene tree with leaf set $\mathbb {G}$ and that $\sigma :\mathbb {G}\rightarrow \mathbb {S}$ and $t:V^0\rightarrow \{\mathfrak {s},\mathfrak {d},\mathfrak {t}\} \cup \{0,1\}$ are the maps described above. Then we say that *S* *is a species tree for* $(T;t,\sigma )$ if there is a map $\mu :V\rightarrow W\cup F$ such that, for all $x\in V$:

1. *Leaf constraint.* If $x\in \mathbb {G}$ then $\mu (x)=\sigma (x)$.
2. *Event constraint.*
  (i) If $$t(x)=\mathfrak {s}$$, then $$\mu (x) = {\text {lca}}_S(\sigma _{T_{\mathcal {\overline{E}}}}(x))$$.
  (ii) If $t(x) \in \{\mathfrak {d}, \mathfrak {t}\}$, then $\mu (x)\in F$.
  (iii) If $t(x)=\mathfrak {t}$ and $(x,y)\in \mathcal {E}$, then $\mu (x)$ and $\mu (y)$ are incomparable in *S*.
3. *Ancestor constraint.* Let $x,y\in V$ with $x\prec _{T_{\mathcal {\overline{E}}}} y$. Note, the latter implies that the path connecting *x* and *y* in *T* does not contain transfer edges. We distinguish two cases:
  (i) If $$t(x),t(y)\in \{\mathfrak {d}, \mathfrak {t}\}$$, then $$\mu (x)\preceq _S \mu (y)$$,
  (ii) otherwise, i.e., at least one of *t*(*x*) and *t*(*y*) is a speciation $\mathfrak {s}$, $\mu (x)\prec _S\mu (y)$.
  We call $\mu$ the *reconciliation map* from $(T;t,\sigma )$ to *S*.

Definition 2 is a natural generalization of the map defined in [10], that is, in the absence of horizontal gene transfer, Condition (M2.iii) vanishes and thus, the proposed reconciliation map precisely coincides with the one given in [10]. In case that the event-labeling of *T* is unknown, but a species tree *S* is given, the authors in [31, 54] gave an axiom set, called DTL-scenario, to reconcile *T* with *S*. This reconciliation is then used to infer the event-labeling *t* of *T*. The “usual” DTL axioms explicitly refer to binary, fully resolved gene and species trees. We therefore use a different axiom set that is, nevertheless, equivalent to DTL-scenarios in case the considered gene trees are binary [63].

Condition (M1) ensures that each leaf of *T*, i.e., an extant gene in $\mathbb {G}$, is mapped to the species in which it resides. Condition (M2.i) and (M2.ii) ensure that each vertex of *T* is either mapped to a vertex or an edge in *S* such that a vertex of *T* is mapped to an interior vertex of *S* if and only if it is a speciation vertex. We will discuss (M2.i) in further detail below. Condition (M2.iii) maps the vertices of a transfer edge in a way that they are incomparable in the species tree and is used to satisfy axiom (O3). Condition (M3) refers only to the connected components of $T_{\mathcal {\overline{E}}}$ and is used to preserve the ancestor order $\preceq _T$ of *T* along the paths that do not contain transfer edges is preserved.

It needs to be discussed, why one *should* map a speciation vertex *x* to ${\text {lca}}_S(\sigma _{T_{\mathcal {\overline{E}}}}(x))$ as required in (M2.i). The next lemma shows, that one *can* put $\mu (x) = {\text {lca}}_S(\sigma _{T_{\mathcal {\overline{E}}}}(x))$.

### **Lemma 4.1**

Nøjgaard et al. [63] Let $\mu$ be a reconciliation map from $(T;t,\sigma )$ to *S* that satisfies (M1) and (M3), then $\mu (u)\succeq _S {\text {lca}}_S(\sigma _{T_{\mathcal {\overline{E}}}}(u))$ for any $u\in V(T)$.

Condition (M2.i) implies in particular the weaker property “(M2.i’) if $t(x)=\mathfrak {s}$ then $\mu (x)\in W$”. In the light of Lemma 4.1, $\mu (x)={\text {lca}}_S(\sigma _{T_{\mathcal {\overline{E}}}}(x))$ is the lowest possible choice for the image of a speciation vertex. Note that there are possibly exponentially many reconciliation maps, whenever $\mu (x)\succ _S{\text {lca}}_S(\sigma _{T_{\mathcal {\overline{E}}}}(x))$ is allowed for speciation vertices *x*. First, we we restrict our attention to those maps that satisfy (M2.i) only. In particular, as we shall see in “Binary gene trees” section, there is a neat characterization of maps that satisfy (M2.i) that does, however, not work for maps with “relaxed” (M2.i), as discussed in “Limitations of informative triples and reconciliation maps” section.

Moreover, we have the following result, which is a mild generalization of [63].

### **Lemma 4.2**

*Let* $\mu$ *be a reconciliation map from a gene tree* $(T;t,\sigma )$ *to S*.

1. If $v,w\in V(T)$ are in the same connected component of $T_{\mathcal {\overline{E}}}$, then $\mu ({\text {lca}}_{T_{\mathcal {\overline{E}}}}(v,w)) \succeq _S {\text {lca}}_S(\mu (v),\mu (w))$.
2. If $(T;t,\sigma )$ is a binary gene tree and *x* a speciation vertex with children *v*, *w* in *T*, then then $\mu (v)$ and $\mu (w)$ are incomparable in *S*.

### *Proof*

Let $v,w\in V(T)$ be in the same connected component of $T_{\mathcal {\overline{E}}}$. Assume that *v* and *w* are comparable in $T_{\mathcal {\overline{E}}}$ and that w.l.o.g. $v\succ _{T_{\mathcal {\overline{E}}}} w$. Condition (M3) implies that $\mu (v)\succeq _S\mu (w)$. Hence, $v = {\text {lca}}_{T_{\mathcal {\overline{E}}}}(v,w)$ and $\mu (v) = {\text {lca}}_S(\mu (v),\mu (w))$ and we are done.

Now assume that *v* and *w* are incomparable in $T_{\mathcal {\overline{E}}}$. Consider the unique path *P* connecting *w* with *v* in $T_{\mathcal {\overline{E}}}$. This path *P* is uniquely subdivided into a path $P'$ and a path $P''$ from ${\text {lca}}_{T_{\mathcal {\overline{E}}}}(v,w)$ to *v* and *w*, respectively. Condition (M3) implies that the images of the vertices of $P'$ and $P''$ under $\mu$, resp., are ordered in *S* with regards to $\preceq _S$ and hence, are contained in the intervals $Q'$ and $Q''$ that connect $\mu ({\text {lca}}_{T_{\mathcal {\overline{E}}}}(v,w))$ with $\mu (v)$ and $\mu (w)$, respectively. In particular, $\mu ({\text {lca}}_{T_{\mathcal {\overline{E}}}}(v,w))$ is the largest element (w.r.t. $\preceq _S$) in the union of $Q'\cup Q''$ which contains the unique path from $\mu (v)$ to $\mu (w)$ and hence also ${\text {lca}}_S(\mu (v),\mu (w))$.

Item 2 was already proven in [63]. $\square$

Assume now that there is a reconciliation map $\mu$ from $(T;t,\sigma )$ to *S*. From a biological point of view, however, it is necessary to reconcile a gene tree with a species tree such that genes do not “travel through time”, a see Fig. 4 for an example.

### **Definition 3**

(Time Map) The map $\tau _T: V(T) \rightarrow \mathbb {R}$ is a time map for the rooted tree *T* if $x\prec _T y$ implies $\tau _T(x)>\tau _T(y)$ for all $x,y\in V(T)$.

### **Definition 4**

A reconciliation map $\mu$ from $(T;t,\sigma )$ to *S* is *time-consistent* if there are time maps $\tau _T$ for *T* and $\tau _S$ for *S* for all $u\in V(T)$ satisfying the following conditions:

1. If $$t(u) \in \{\mathfrak {s}, \odot \}$$, then $$\tau _T(u) = \tau _S(\mu (u))$$.
2. If $t(u)\in \{\mathfrak {d},\mathfrak {t}\}$ and, thus $\mu (u)=(x,y)\in E(S)$, then $\tau _S(y)>\tau _T(u)>\tau _S(x)$.

Condition (T1) is used to identify the time-points of speciation vertices and leaves *u* in the gene tree with the time-points of their respective images $\mu (u)$ in the species trees. Moreover, duplication or HGT vertices *u* are mapped to edges $\mu (u)=(x,y)$ in *S* and the time point of *u* must thus lie between the time points of *x* and *y* which is ensured by Condition (T2). Nøjgaard et al. [63] designed an $\mathcal {O}(|V(T)|\log (|V(S)|))$-time algorithm to check whether a given reconciliation map $\mu$ is time-consistent, and an algorithm with the same time complexity for the construction of a time-consistent reconciliation map, provided one exists. Clearly, a necessary condition for the existence of time-consistent reconciliation maps from $(T;t,\sigma )$ to *S* is the existence of *some* reconciliation map from $(T;t,\sigma )$ to *S*. In the next section, we first characterize the existence of reconciliation maps and discuss open time-consistency problems.

## From gene trees to species trees

**Fig. 2:**
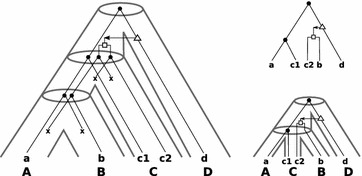
*Left* an example of a “true” history of a gene tree that evolves along the (*tube-like*) species tree (taken from [11]). The set of extant genes $\mathbb {G}$ comprises $a,b,c_1,c_2$ and *d* and $\sigma$ maps each gene in $\mathbb {G}$ to the species (*capitals below the genes*) $A,B,C,D\in \mathbb {S}$. *Upper right* the observable gene tree $(T;t,\sigma )$ is shown. To derive $\mathcal {S}(T;t,\sigma )$ we cannot use the triples $\mathcal {R}_0(T)$, that is, we need to remove the transfer edges. To be more precise, if we would consider $\mathcal {R}_0(T)$ we obtain the triples $\mathsf {(ac_1|d)}$ and $\mathsf {(c_2d|a)}$ which leads to the two contradicting species triples $\mathsf {(AC|D)}$ and $\mathsf {(CD|A)}$. Thus, we restrict $\mathcal {R}_0$ to $T_{\mathcal {\overline{E}}}$ and obtain $\mathcal {R}_0(T_{\mathcal {\overline{E}}}) = \{\mathsf {(ac_1|d)}\}$. However, this triple alone would not provide enough information to obtain a species tree such that a valid reconciliation map $\mu$ can be constructed. Hence, we take $\mathcal {R}_1(T_{\mathcal {\overline{E}}})=\{\mathsf {(bc_2|d)}\}$ into account and obtain $\mathcal {S}(T;t,\sigma ) = \{\mathsf {(AC|D)},\mathsf {(BC|D)}\}$. *Lower right* a least resolved species tree *S* (obtained with BUILD) that displays all triples in $\mathcal {S}(T;t,\sigma )$ together with the reconciled gene tree $(T;t,\sigma )$ is shown. Although *S* does not display the triple $\mathsf {(AB|C)}$ as in the true history, this tree *S* does not pretend a higher resolution than actually supported by $(T;t,\sigma )$. Clearly, as more gene trees (gene families) are available as more information about the resolution of the species tree can be provided

Since a gene tree *T* is uniquely determined by its induced triple set $\mathcal {R}(T)$, it is reasonable to expect that a lot of information on the species tree(s) for $(T;t, \sigma )$ is contained in the images of the triples in $\mathcal {R}(T)$, or more precisely their leaves under $\sigma$. However, not all triples in $\mathcal {R}(T)$ are informative, see Fig. 2 for an illustrative example. In the absence of HGT, it has already been shown by Hernandez-Rosales et al. [10] that the informative triples $r\in \mathcal {R}(T)$ are precisely those that are rooted at a speciation event and where the genes in *r* reside in three distinct species. However, in the presence of HGT we need to further subdivide the informative triples as follows.

### **Definition 5**

Let $(T;t,\sigma )$ be a given event-labeled gene tree with respective set of transfer-edges $\mathcal {E}= \{e_1,\dots ,e_h\}$ and $T_{\mathcal {\overline{E}}}$ as defined above. We define

$$\begin{aligned} \mathcal {R}_{\sigma }(T_{\mathcal {\overline{E}}}) =& \{\mathsf {(ab|c)} \in \mathcal {R}(T_{\mathcal {\overline{E}}}) :\sigma (a),\sigma (b),\sigma (c)&\quad \\&\text {are pairwise distinct} \} \end{aligned}$$

as the subset of all triples displayed in $T_{\mathcal {\overline{E}}}$ such that the leaves are from pairwise distinct species.

Let

$$\begin{aligned} \mathcal {R}_0(T_{\mathcal {\overline{E}}}) :=\{\mathsf {(ab|c)} \in \mathcal {R}_{\sigma }(T_{\mathcal {\overline{E}}}) :t({\text {lca}}_{T_{\mathcal {\overline{E}}}}(a,b,c)) = \mathfrak {s}\} \end{aligned}$$

be the set of triples in $\mathcal {R}_{\sigma }(T_{\mathcal {\overline{E}}})$ that are rooted at a speciation event.

For each $e_i=(x,y) \in \mathcal {E}$ define

$$\begin{aligned} \begin{array}{ll} \mathcal {R}_i(T_{\mathcal {\overline{E}}}) :=\{ \mathsf {(ab|c)} :&{} \sigma (a),\sigma (b),\sigma (c) \text { are pairwise distinct} \\ &{}\text {and either } a,b\in L_{T_{\mathcal {\overline{E}}}}(x), c\in L_{T_{\mathcal {\overline{E}}}}(y) \\ &{}\text {or } c\in L_{T_{\mathcal {\overline{E}}}}(x), a,b\in L_{T_{\mathcal {\overline{E}}}}(y) \}. \end{array} \end{aligned}$$

Hence, $\mathcal {R}_i(T_{\mathcal {\overline{E}}})$ contains a triple $\mathsf {(ab|c)}$ for every $a,b\in L_{T_{\mathcal {\overline{E}}}}(x), c\in L_{T_{\mathcal {\overline{E}}}}(y)$ that reside in pairwise distinct species. Analogously, for any $a,b\in L_{T_{\mathcal {\overline{E}}}}(y), c\in L_{T_{\mathcal {\overline{E}}}}(x)$ there is a triple $\mathsf {(ab|c)}\in \mathcal {R}_i(T_{\mathcal {\overline{E}}})$, if $\sigma (a),\sigma (b),\sigma (c)$ are pairwise distinct.

The *informative triples* of *T* are comprised in the set $\mathcal {R}(T;t,\sigma ) = \cup _{i=0}^h \mathcal {R}_i(T_{\mathcal {\overline{E}}})$.

Finally, we define the informative species triple set

$$\begin{aligned} \mathcal {S}(T;t,\sigma ):=\{\mathsf {(\sigma (a)\sigma (b)|\sigma (c))} :\mathsf {(ab|c)} \in \mathcal {R}(T;t,\sigma ) \} \end{aligned}$$

that can be inferred from the informative triples of $(T;t,\sigma )$.

### Binary gene trees

In this section, we will be concerned only with binary, i.e., “fully resolved” gene trees, if not stated differently. This is justified by the fact that a speciation or duplication event instantaneously generates exactly two offspring. However, we will allow also non-binary species tree to model incomplete knowledge of the exact species phylogeny. Non-binary gene trees are discussed in “Non-binary gene trees” section.

Hernandez et al. [10] established the following characterization for the HGT-free case.

#### **Theorem 5.1**

For a given gene tree $(T;t, \sigma )$ on $\mathbb {G}$ that *does not* contain HGT and $\mathfrak {S}:=\{\mathsf {(\sigma (a)\sigma (b)|\sigma (c))} :\mathsf {(ab|c)} \in \mathcal {R}_0(T)\}$, the following statement is satisfied:

There is a species tree on $\mathbb {S}= \sigma (\mathbb {G})$ for $(T;t, \sigma )$ if and only if the triple set $\mathfrak {S}$ is consistent.

**Fig. 3:**
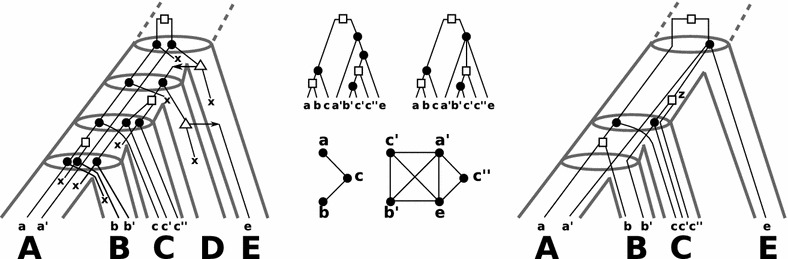
Consider the “true” history (*left*) that is also shown in Fig. 1. The center-left gene tree $(T;t,\sigma )$ is biologically feasible and obtained as the observable part of the true history. There is no reconciliation map for $(T;t,\sigma )$ to any species tree according to Def. 2 because $\mathcal {S}(T;t,\sigma )$ is inconsistent (cf. Thm. 5.4). The graph in the lower-center depicts the orthology-relation that comprises all pairs (*x*, *y*) of vertices for which $t({\text {lca}}(x,y)) =\mathfrak {s}$. The center-right gene tree $(T';t,\sigma )$ is non-binary and can directly be computed from the orthology-relation. Although $\mathcal {S}(T';t,\sigma )$ is inconsistent, there is a valid reconciliation map $\mu$ to a species tree for $(T';t,\sigma )$ according to Def. 2 (*right*). Note, both trees $(T;t,\sigma )$ and $(T';t,\sigma )$ satisfy axioms (O1)–(O3) and even (O3.A). However, the reconciliation map $\mu$ does not satisfy the extra Condition (M2.iv), since $\mu (z)$ and $\mu (a')=A$ are comparable, although *z* and $a'$ are children of a common speciation vertex. Therefore, Axioms (O1)–(O3) and (O3.A) do not imply (M2.iv). Moreover, Thm. 5.7 implies that there is no restricted reconciliation map for $(T;t,\sigma )$ as well as $(T';t,\sigma )$ and any species tree, since $\mathcal {S}(T;t,\sigma )$ and $\mathcal {S}(T';t,\sigma )$ are inconsistent. See text for further details

We emphasize that the results established in [10] are only valid for binary gene trees, although this was not explicitly stated. For an example that shows that Theorem 5.1 is not always satisfied for non-binary gene trees see Fig. 3. Lafond and El-Mabrouk [12, 48] established a similar result as in Theorem 5.1 by using only species triples that can be obtained directly from a given orthology/paralogy-relation. However, they require a stronger version of axiom (O3.a), that is, the images of all children of a speciation vertex must be pairwisely incomparable in the species tree. We, too, will use this restriction in “Non-binary gene trees” section.

In what follows, we generalize the latter result and show that consistency of $\mathcal {S}(T;t,\sigma )$ characterizes whether there is a species tree *S* for $(T;t,\sigma )$ even if $(T;t,\sigma )$ contains HGT.

#### **Lemma 5.2**

If $\mu$ *is a reconciliation map from a gene tree* $(T;t,\sigma )$ *to a species tree* *S* and $\mathsf {(ab|c)} \in \mathcal {R}(T;t,\sigma )$ *, then* $\mathsf {(\sigma (a)\sigma (b)|\sigma (c))}$ *is displayed in* *S*.

#### *Proof*

Recall that $\mathbb {G}$ is the leaf set of $T=(V,E)$ and, by Lemma 3.1, of $T_{\mathcal {\overline{E}}}$. Let $$\{a,b,c\} \in \left( {\begin{array}{c}\mathbb {G}\\ 3\end{array}}\right)$$ and assume w.l.o.g. $\mathsf {(ab|c)} \in \mathcal {R}(T;t,\sigma )$.

First assume that $\mathsf {(ab|c)} \in \mathcal {R}_0$, that is $\mathsf {(ab|c)}$ is displayed in $T_{\mathcal {\overline{E}}}$ and $t({\text {lca}}_{T_{\mathcal {\overline{E}}}}(a,b,c)) = \mathfrak {s}$. For simplicity set $u={\text {lca}}_{T_{\mathcal {\overline{E}}}}(a,b,c)$ and let *x*, *y* be its children in $T_{\mathcal {\overline{E}}}$. Since $\mathsf {(ab|c)} \in \mathcal {R}_0$, we can assume that w.l.o.g. $a,b\in L_{T_{\mathcal {\overline{E}}}}(x)$ and $c\in L_{T_{\mathcal {\overline{E}}}}(y)$. Hence, $x\succeq _{T_{\mathcal {\overline{E}}}} {\text {lca}}_{T_{\mathcal {\overline{E}}}}(a,b)$ and $y\succeq _{T_{\mathcal {\overline{E}}}} c$. Condition (M3) implies that $\mu (y)\succeq _S \mu (c) = \sigma (c)$. Moreover, Condition (M3) and Lemma 4.2(1) imply that $\mu (x)\succeq _S \mu ({\text {lca}}_{T_{\mathcal {\overline{E}}}}(a,b)) \succeq _S {\text {lca}}_S(\mu (a),\mu (b)) = {\text {lca}}_S(\sigma (a),\sigma (b))$. Since $t(u)=\mathfrak {s}$, we can apply Lemma 4.2(2) and conclude that $\mu (x)$ and $\mu (y)$ are incomparable in *S*. Hence, $\sigma (c)$ and ${\text {lca}}_S(\sigma (a),\sigma (b))$ are incomparable. Thus, the triple $\mathsf {(\sigma (a)\sigma (b)|\sigma (c))}$ must be displayed in *S*.

Now assume that $\mathsf {(ab|c)} \in \mathcal {R}_i$ for some transfer edge $e_i = (x,y)\in \mathcal {E}$. For $e_i = (x,y)$ we either have $a,b\in L_{T_{\mathcal {\overline{E}}}}(x)$ and $c\in L_{T_{\mathcal {\overline{E}}}}(y)$ or $c\in L_{T_{\mathcal {\overline{E}}}}(x)$ and $a,b\in L_{T_{\mathcal {\overline{E}}}}(y)$. W.l.o.g. let $a,b\in L_{T_{\mathcal {\overline{E}}}}(x)$ and $c\in L_{T_{\mathcal {\overline{E}}}}(y)$. Thus, $x\succeq _{T_{\mathcal {\overline{E}}}} {\text {lca}}_{T_{\mathcal {\overline{E}}}}(a,b)$ and $y\succeq _{T_{\mathcal {\overline{E}}}} c$. Condition (M3) implies that $\mu (y)\succeq _S \mu (c) = \sigma (c)$. Moreover, Condition (M3) and Lemma 4.2(1) imply that $\mu (x)\succeq _S \mu ({\text {lca}}_{T_{\mathcal {\overline{E}}}}(a,b)) \succeq _S {\text {lca}}_S(\mu (a),\mu (b)) = {\text {lca}}_S(\sigma (a),\sigma (b))$. Since $t(x)=\mathfrak {t}$, we can apply (M2.iii) and conclude that $\mu (x)$ and $\mu (y)$ are incomparable in *S*. Hence, $\sigma (c)$ and ${\text {lca}}_S(\sigma (a),\sigma (b))$ are incomparable. Thus, the triple $\mathsf {(\sigma (a)\sigma (b)|\sigma (c))}$ must be displayed in *S*. $\square$

#### **Lemma 5.3**

*Let* $S=(W,F)$ *be a species tree on* $\mathbb {S}$ *. Then there is a reconciliation map* $\mu$ *from a gene tree* $(T;t,\sigma )$ *to S whenever S displays all triples in* $\mathcal {S}(T;t,\sigma )$.

#### *Proof*

Recall that $\mathbb {G}$ is the leaf set of $T=(V,E)$ and, by Lemma 3.1, of $T_{\mathcal {\overline{E}}}$. In what follows, we write $\mathcal {L}(u)$ instead of the more complicated writing $L_{T_{\mathcal {\overline{E}}}}(u)$ and, for consistency and simplicity, we also often write $\sigma (\mathcal {L}(u))$ instead of $\sigma _{T_{\mathcal {\overline{E}}}}(u)$. Put $S=(W,F)$ and $\mathcal {S} = \mathcal {S}(T;t,\sigma )$. We first consider the subset $U=\{x\in V \mid x\in \mathbb {G}\text { or } t(x) = \mathfrak {s}\}\}$ of *V* comprising the leaves and speciation vertices of *T*.

In what follows we will explicitly construct $\mu : V \rightarrow W\cup F$ and verify that $\mu$ satisfies Conditions (M1), (M2) and (M3). To this end, we first set for all $x\in U$:

1. $$\mu (x) = \sigma (x)$$, if $$x\in \mathbb {G}$$,
2. $$\mu (x)= {\text {lca}}_S(\sigma (\mathcal {L}(x)))$$, if $$t(x)=\mathfrak {s}$$.

Conditions (S1) and (M1), as well as (S2) and (M2.i) are equivalent.

For later reference, we show that ${\text {lca}}_S(\sigma (\mathcal {L}(x))) \in W^0 = W\setminus \mathbb {S}$ and that there are two leaves $a,b\in \mathcal {L}(x)$ such that $\sigma (a) \ne \sigma (b)$, whenever $t(x)=\mathfrak {s}$. By Condition (O3.a), for the two children *v* and *w* of *x* in *T* we have $\sigma (\mathcal {L}(v)) \cap \sigma (\mathcal {L}(w)) = \emptyset$. Moreover, Lemma 3.1 implies that both $\mathcal {L}(v)$ and $\mathcal {L}(w)$ are non-empty subsets of $\mathbb {G}$ and hence, neither $\sigma (\mathcal {L}(v))=\emptyset$ nor $\sigma (\mathcal {L}(w))=\emptyset$. Thus, there are two leaves $a, b\in \mathcal {L}(x)$ such that $\sigma (a) \ne \sigma (b)$. Hence, ${\text {lca}}_S(\sigma (\mathcal {L}(x))) \in W^0 = W\setminus \mathbb {S}$.

**Claim 1:** *For all* $x,y\in U$ *with* $x\prec _{T_{\mathcal {\overline{E}}}} y$ *we have* $\mu (x)\prec _S \mu (y)$.

Note, *y* must be an interior vertex, since $x\prec _{T_{\mathcal {\overline{E}}}} y$.

Hence $$t(y)=\mathfrak {s}$$.

If *x* is a leaf, then $\mu (x)=\sigma (x)\in \mathbb {S}$. As argued above, $\mu (y) \in W\setminus \mathbb {S}$. Since $x\in \mathcal {L}(y)$ and $\sigma (\mathcal {L}(y))\ne \emptyset$, we have $\sigma (x) \in \sigma (\mathcal {L}(y))\subseteq \mathbb {S}$ and thus, $\mu (x)\prec _S \mu (y)$.

Now assume that *x* is an interior vertex and hence, $t(x)=\mathfrak {s}$. Again, there are leaves $a,b \in \mathcal {L}(x)$ with $A = \sigma (a)\ne \sigma (b)=B$. Since $t(y)=\mathfrak {s}$, vertex *y* has two children in $T_{\mathcal {\overline{E}}}$. Let $y'$ denote the child of *y* with $x\preceq _{T_{\mathcal {\overline{E}}}} y'$. Since $\mathcal {L}(x)\subseteq \mathcal {L}(y')\subsetneq \mathcal {L}(y)$, we have $\mathcal {L}(y)\setminus \mathcal {L}(y')\ne \emptyset$ and, by Condition (O3.a), there is a gene $c\in \mathcal {L}(y)\setminus \mathcal {L}(y') \subseteq \mathcal {L}(y)\setminus \mathcal {L}(x)$ with $\sigma (c)=C\ne A,B$. By construction, $\mathsf {(ab|c)}\in \mathcal {R}_0$ and hence, $\mathsf {(AB|C)}\in \mathcal {S}(T;t,\sigma )$. Hence, ${\text {lca}}_S(A,B)\prec _S {\text {lca}}_S(A,B,C)$. Since this holds for all triples $\mathsf {(x'x''|z)}$ with $x',x''\in \mathcal {L}(x)$ and $z\in \mathcal {L}(y)\setminus \mathcal {L}(y')$, we can conclude that

$$\begin{aligned} \mu (x)&= {\text {lca}}_S(\sigma (\mathcal {L}(x))) \\&\prec _S {\text {lca}}_S(\sigma (\mathcal {L}(x))\cup \sigma (\mathcal {L}(y) \setminus \mathcal {L}(y'))). \end{aligned}$$

Since $\sigma (\mathcal {L}(x))\cup \sigma (\mathcal {L}(y) \setminus \mathcal {L}(y')) \subseteq \sigma (\mathcal {L}(y))$ we obtain

$$\begin{aligned} & {\text{lca}}_S(\sigma (\mathcal {L}(x))\cup \sigma (\mathcal {L}(y) \setminus \mathcal {L}(y^{\prime}))) \\ & \quad \preceq _S {\text{lca}}_S(\sigma (\mathcal {L}(y))) = \mu (y). \end{aligned}$$

Hence, $\mu (x)\prec _S\mu (y)$. Thus, the claim is proven. $\square$

We continue to extend $\mu$ to the entire set *V*. To this end, observe first that if $t(x) \in \{\mathfrak {t}, \mathfrak {d}\}$ then we wish to map *x* on an edge $\mu (x) = (u,v) \in F$ such that Lemma 4.1 is satisfied: $v\succeq _S {\text {lca}}_S(\sigma (\mathcal {L}(x)))$. Such an edge exists for $v = {\text {lca}}_S(\sigma (\mathcal {L}(x)))$ in *S* by construction. Every speciation vertex *y* with $y\succ _{T_{\mathcal {\overline{E}}}} x$ therefore necessarily maps on the vertex *u* or above, i.e., $\mu (y) \succeq _S u$ must hold. Thus, we set:

(S3) $$\mu (x) = (u,{\text {lca}}_S(\sigma (\mathcal {L}(x))))$$, if $$t(x)\in \{\mathfrak {t}, \mathfrak {d}\}$$,

which now makes $\mu$ a map from *V* to $W\cup F$.

By construction of $\mu$, Conditions (M1), (M2.i), (M2.ii) are satisfied by $\mu$.

We proceed to show that (M3) is satisfied.

**Claim 2:** *For all* $x,y\in V$ *with* $x\prec _{T_{\mathcal {\overline{E}}}} y$, *Condition (M3) is satisfied.*

If both *x* and *y* are speciation vertices, then we can apply the Claim 1 to conclude that $\mu (x)\prec _S \mu (y)$. If *x* is a leaf, then we argue similarly as in the proof of Claim 1 to conclude that $\mu (x)\preceq _S \mu (y)$.

Now assume that both *x* and *y* are interior vertices of *T* and at least one vertex of *x*, *y* is not a speciation vertex. Since, $x\prec _{T_{\mathcal {\overline{E}}}} y$ we have $\mathcal {L}(x) \subseteq \mathcal {L}(y)$ and thus, $\sigma (\mathcal {L}(x)) \subseteq \sigma (\mathcal {L}(y))$.

We start with the case $t(y)=\mathfrak {s}$ and $t(x)\in \{\mathfrak {d}, \mathfrak {t}\}$. Since $t(y)=\mathfrak {s}$, vertex *y* has two children in $T_{\mathcal {\overline{E}}}$. Let $y'$ be the child of *y* with $x\preceq _{T_{\mathcal {\overline{E}}}} y'$. If $\sigma (\mathcal {L}(x))$ contains only one species *A*, then $\mu (x) = (u,A)\prec _S u\preceq _S {\text {lca}}_S(\sigma (\mathcal {L}(y))) = \mu (y)$. If $\sigma (\mathcal {L}(x))$ contains at least two species, then there are $a,b\in \mathcal {L}(x)$ with $\sigma (a)=A\ne \sigma (b)=B$ Moreover, since $\mathcal {L}(x)\subseteq \mathcal {L}(y')\subsetneq \mathcal {L}(y)$, we have $\mathcal {L}(y)\setminus \mathcal {L}(y')\ne \emptyset$ and, by Condition (O3.a), there is a gene $c\in \mathcal {L}(y)\setminus \mathcal {L}(y') \subseteq \mathcal {L}(y)\setminus \mathcal {L}(x)$ with $\sigma (c)=C\ne A,B$. By construction, $\mathsf {(ab|c)}\in \mathcal {R}_0$ and hence $\mathsf {(AB|C)}\in \mathcal {S}(T;t,\sigma )$. Now we can argue similar as in the proof of the Claim 1, to see that

$$\begin{aligned} \mu (x)&= (u,{\text {lca}}_S(\sigma (\mathcal {L}(x)))) \prec _S u \\&\preceq _S {\text {lca}}_S(\sigma (\mathcal {L}(y))) = \mu (y). \end{aligned}$$

If $t(x)=\mathfrak {s}$ and $t(y)\in \{\mathfrak {d}, \mathfrak {t}\}$, then $\sigma (\mathcal {L}(x)) \subseteq \sigma (\mathcal {L}(y))$ implies that

$$\begin{aligned} \mu (x)&= {\text {lca}}_S(\sigma (\mathcal {L}(x)))\preceq _S {\text {lca}}_S(\sigma (\mathcal {L}(y))) \\&\prec _S(u,{\text {lca}}_S(\sigma (\mathcal {L}(y)))) = \mu (y). \end{aligned}$$

Finally assume that $t(x),t(y)\in \{\mathfrak {d}, \mathfrak {t}\}$. If $\sigma (\mathcal {L}(x)) = \sigma (\mathcal {L}(y))$, then $\mu (x) = \mu (y)$. Now let $\sigma (\mathcal {L}(x)) \subsetneq \sigma (\mathcal {L}(y))$ which implies that ${\text {lca}}_S(\sigma (\mathcal {L}(x)))\preceq _S {\text {lca}}_S(\sigma (\mathcal {L}(y)))$. If ${\text {lca}}_S(\sigma (\mathcal {L}(x))) = {\text {lca}}_S(\sigma (\mathcal {L}(y)))$, then $\mu (x) = \mu (y)$. If ${\text {lca}}_S(\sigma (\mathcal {L}(x)))\prec _S {\text {lca}}_S(\sigma (\mathcal {L}(y)))$, then

$$\begin{aligned} \mu (x)&=(u,{\text {lca}}_S(\sigma (\mathcal {L}(x)))) \prec _S u \\&\preceq _S {\text {lca}}_S(\sigma (\mathcal {L}(y))) \prec (u',{\text {lca}}_S(\sigma (\mathcal {L}(y)))) \\&=\mu (y). \end{aligned}$$

It remains to show (M2.iii), that is, if $e_i=(x,y)$ is a transfer-edge, then $\mu (x)$ and $\mu (y)$ are incomparable in *S*. Since (*x*, *y*) is a transfer edge and by Condition (O3.b), $\sigma (\mathcal {L}(x)) \cap \sigma (\mathcal {L}(y)) = \emptyset$. If $\sigma (\mathcal {L}(x))=\{A\}$ and $\sigma (\mathcal {L}(y))=\{C\}$, then $\mu (x) = (u,A)$ and $\mu (y) = (u',C)$. Since *A* and *C* are distinct leaves in *S*, $\mu (x)$ and $\mu (y)$ are incomparable. Assume that $|\sigma (\mathcal {L}(x))|>1$. Hence, there are leaves $a,b \in \mathcal {L}(x)$ with $A = \sigma (a)\ne \sigma (b)=B$ and $c\in \mathcal {L}(y)$ with $\sigma (c)=C\ne A,B$. By construction, $\mathsf {(ab|c)}\in \mathcal {R}_i$ and hence, $\mathsf {(AB|C)}\in \mathcal {S}(T;t,\sigma )$. The latter is fulfilled for all triples $\mathsf {(x'x''|c)}\in \mathcal {R}_i$ with $x',x''\in \mathcal {L}(x)$, and, therefore, ${\text {lca}}_S(\sigma (\mathcal {L}(x))\cup \{C\}) \succ _S {\text {lca}}_S(\sigma (\mathcal {L}(x)))$. Set $v={\text {lca}}_S(\sigma (\mathcal {L}(x))\cup \{C\})$. Thus, there is an edge $(v,v')$ in *S* with $v'\succeq _S {\text {lca}}_S(\sigma (\mathcal {L}(x)))$ and an edge $(v,v'')$ such that $v''\succeq _S C$. Hence, either $\mu (x) = (v,v')$ or $\mu (x) = (u,{\text {lca}}_S(\sigma (\mathcal {L}(x)))$ and $v'\succeq _S u$. Assume that $\sigma (\mathcal {L}(y))$ contains only the species *C* and thus, $\mu (y) = (u',C)$. Since $v''\succeq _S C$, we have either $v'' = C$ which implies that $\mu (y) = (v,v'')$ or $v'' \succ _S C$ which implies that $\mu (y) = (u',C)$ and $v''\succeq _S u'$. Since both vertices $v'$ and $v''$ are incomparable in *S*, so $\mu (x)$ and $\mu (y)$ are. If $|\sigma (\mathcal {L}(y))|>1$, then we set $v={\text {lca}}_S(\sigma (\mathcal {L}(x))\cup \sigma (\mathcal {L}(y)))$ and we can argue analogously as above and conclude that there are edges $(v,v')$ and $(v,v'')$ in *S* such that $v'\succeq _S {\text {lca}}_S(\sigma (\mathcal {L}(x)))$ and $v''\succeq _S {\text {lca}}_S(\sigma (\mathcal {L}(y)))$. Again, since $v'$ and $v''$ are incomparable in *S* and by construction of $\mu$, $\mu (x)$ and $\mu (y)$ are incomparable. Thus, the claim is proven. $\square$

Lemma 5.2 implies that consistency of the triple set $\mathcal {S}(T; t,\sigma )$ is necessary for the existence of a reconciliation map from $(T; t,\sigma )$ to a species tree on $\mathbb {S}$. Lemma 5.3, on the other hand, establishes that this is also sufficient. Thus, we have

#### **Theorem 5.4**

*There is a species tree on* $\mathbb {S}= \sigma (\mathbb {G})$ *for a binary gene tree* $(T;t, \sigma )$ *on* $\mathbb {G}$ *if and only if the triple set* $\mathcal {S}(T; t,\sigma )$ *is consistent.*

### Non-binary gene trees

Now, we consider arbitrary, possibly non-binary gene trees that might be used to model incomplete knowledge of the exact genes phylogeny. Consider the “true” history of a gene tree that evolves along the (tube-like) species tree in Fig. 3 (left). The observable gene tree $(T;t,\sigma )$ is shown in Fig. 3 (center-left). Since $\mathsf {(ab|c)},\mathsf {(b'c'|a')} \in \mathcal {R}_0$, we obtain a set of species triples $\mathcal {S}(T;t,\sigma )$ that contain the pair of inconsistent species triple $\mathsf {(AB|C)},\mathsf {(BC|A)}$. Thus, there is no reconciliation map for $(T;t,\sigma )$ and any species tree, although $(T;t,\sigma )$ is biologically feasible. Consider now the “orthology” graph *G* (shown below the gene trees) that has as vertex set $\mathbb {G}$ and two genes *x*, *y* are connected by an edge if ${\text {lca}}(x,y)$ is a speciation vertex. Such graphs can be obtained from orthology inference methods [14, 36–38] and the corresponding *non-binary* gene tree $(T';t,\sigma )$ (center-right) is constructed from such estimates (see [5–7] for further details). Still, we can see that $\mathcal {S}(T';t,\sigma )$ contains the two inconsistent species triples $\mathsf {(AB|C)},\mathsf {(BC|A)}$. However, there is a reconciliation map $\mu$ according to Definition 2 and a species tree *S*, as shown in Fig. 3 (right). Thus, consistency of $\mathcal {S}(T';t,\sigma )$ does not characterize whether there is a valid reconciliation map for non-binary gene trees.

In order to obtain a similar result as in Theorem 5.4 for non-binary gene trees we have to strengthen observability axiom (O3.a) to

(O3.A) If *x* is a speciation vertex with children $v_1,\dots ,v_k$, then $\sigma _{T_{\mathcal {\overline{E}}}}(v_i) \cap \sigma _{T_{\mathcal {\overline{E}}}}(v_j) =\emptyset$, $1\le i<j\le k$;

and to add an extra event constraint to Definition 2:

(M2.iv) Let $v_1,\dots ,v_k$ be the children of the speciation vertex *x*. Then, $\mu (v_i)$ and $\mu (v_j)$ are incomparable in *S*, $1\le i<j\le k$.

We call a reconciliation map that additionally satisfies (M2.iv) a *restricted reconciliation map*. Such restricted reconciliation maps satisfy the condition as required in [12, 48] for the HGT-free case. It can be shown that restricted reconciliation maps imply Condition (O3.A), however, the converse is not true in general, see Fig. 3. Hence, we cannot use the axioms (O1)-(O3) and (O3.A) to derive Condition (M2.iv)—similar to Lemma 4.2(2)—and thus, need to claim it.

In particular, Condition (M2.iv) forbids ancestral relationships of the images $\mu (v_i)$ and $\mu (v_i)$ in *S* for any two distinct children $v_i$ and $v_j$ of a speciation vertex *x*. In Fig. 3 (right) a map $\mu$ is shown that violates Condition (M2.iv). Here, the images $\mu (z)$ and $\mu (a')$ are comparable. The latter might happen, if there are unrecognized HGT events followed by a loss. Condition (M2.iv) is a quite strong restriction, however, it is indispensable for the characterization of reconciliation maps for non-binary gene trees in terms of informative triples, as we shall see soon.

It is now straightforward to obtain the next result.

#### **Lemma 5.5**

*If* $\mu$ *is a restricted reconciliation map from* $(T;t,\sigma )$ *to S and* $\mathsf {(ab|c)} \in \mathcal {R}(T;t,\sigma )$ *, then* $\mathsf {(\sigma (a)\sigma (b)|\sigma (c))}$ *is displayed in S*.

#### *Proof*

Let $$\{a,b,c\} \in \left( {\begin{array}{c}\mathbb {G}\\ 3\end{array}}\right)$$ and assume w.l.o.g. $\mathsf {(ab|c)} \in \mathcal {R}(T;t,\sigma )$.

First assume that $\mathsf {(ab|c)} \in \mathcal {R}_0$, that is $\mathsf {(ab|c)}$ is displayed in $T_{\mathcal {\overline{E}}}$ and $t({\text {lca}}_{T_{\mathcal {\overline{E}}}}(a,b,c)) = \mathfrak {s}$. For simplicity set $u={\text {lca}}_{T_{\mathcal {\overline{E}}}}(a,b,c)$. Hence, there are two children *x*, *y* of *u* in $T_{\mathcal {\overline{E}}}$ such that w.l.o.g. $a,b\in L_{T_{\mathcal {\overline{E}}}}(x)$ and $c\in L_{T_{\mathcal {\overline{E}}}}(y)$. Now we can argue analogously as in the proof of Lemma 5.2 after replacing “we can apply Lemma 4.2(2)” by “we can apply Condition (M2.iv)”. The proof for $\mathsf {(ab|c)} \in \mathcal {R}_i$ remains the same as in Lemma 5.2. $\square$

#### **Lemma 5.6**

*Let S be a species tree on* $\mathbb {S}$ *. Then, there is a restricted reconciliation map* $\mu$ *from a gene tree* $(T;t,\sigma )$ *that satisfies also (O3.A) to S whenever S displays all triples in* $\mathcal {S}(T;t,\sigma )$.

#### *Proof*

The proof is similar to the proof of Lemma 5.6. However, note that a speciation vertex might have more than two children. In these cases, one simply has to apply Axiom (O3.A) instead of Lemma (O3.a) to conclude that (M1), (M2.i)–(M2.iii), (M3) are satisfied.

It remains to show that (M2.iv) is satisfied. To this end, let *x* be a speciation vertex in *T* and the set of its children $\mathsf {Ch}(x) = \{v_1,\dots ,v_k\}$. By axiom (O3.A) we have $\sigma _{T_{\mathcal {\overline{E}}}}(v_i) \cap \sigma _{T_{\mathcal {\overline{E}}}}(v_j) =\emptyset$ for all $i\ne j$. Consider the following partition of $\mathsf {Ch}(x)$ into $\mathsf {Ch}_1$ and $\mathsf {Ch}_2$ that contain all vertices $v_i$ with $|\sigma _{T_{\mathcal {\overline{E}}}}(v_i)|=1$ and $|\sigma _{T_{\mathcal {\overline{E}}}}(v_i)|>1$, respectively. By construction of $\mu$, for all vertices in $v_i,v_j\in \mathsf {Ch}_1$, $i\ne j$ we have that $\mu (v_i)\in \{\sigma (v_i), (u,\sigma (v_i)) \}$ and $\mu (v_j)\in \{\sigma (v_j), (u',\sigma (v_j)) \}$ are incomparable. Now let $v_i\in \mathsf {Ch}_1$ and $v_j\in \mathsf {Ch}_2$. Thus, there are $A,B\in \sigma _{T_{\mathcal {\overline{E}}}}(v_j)$ and $\sigma (v_i)=C$. Hence, $\mathsf {(AB|C)} \in \mathcal {S}(T;t,\sigma )$ Therefore, ${\text {lca}}_S(A,B)$ must be incomparable to *C* in *S*. Since the latter is satisfied for all species in $\sigma _{T_{\mathcal {\overline{E}}}}(v_j)$, ${\text {lca}}_S( \sigma _{T_{\mathcal {\overline{E}}}}(v_j))$ and *C* must be incomparable in *S*. Again, by construction of $\mu$, we see that $\mu (v_i)\in \{C, (u,C) \}$ and $\mu (v_j)\in \{{\text {lca}}_S( \sigma _{T_{\mathcal {\overline{E}}}}(v_j)), (u',{\text {lca}}_S( \sigma _{T_{\mathcal {\overline{E}}}}(v_j))) \}$ are incomparable in *S*. Analogously, if $v_i,v_j\in \mathsf {Ch}_2$, $i\ne j$, then all triples $\mathsf {(AB|C)}$ and $\mathsf {(CD|A)}$ for all $A,B\in \sigma _{T_{\mathcal {\overline{E}}}}(v_j)$ and $C,D\in \sigma _{T_{\mathcal {\overline{E}}}}(v_j)$ are contained in $\mathcal {S}(T;t,\sigma )$ and thus, displayed by *S*. Hence, ${\text {lca}}_S( \sigma _{T_{\mathcal {\overline{E}}}}(v_i))$ and ${\text {lca}}_S( \sigma _{T_{\mathcal {\overline{E}}}}(v_j))$ must be incomparable in *S*. Again, by construction of $\mu$, we obtain that $\mu (v_i)\in \{{\text {lca}}_S( \sigma _{T_{\mathcal {\overline{E}}}}(v_i)), (u,{\text {lca}}_S( \sigma _{T_{\mathcal {\overline{E}}}}(v_i))) \}$ and $\mu (v_j)\in \{{\text {lca}}_S( \sigma _{T_{\mathcal {\overline{E}}}}(v_j)), (u',{\text {lca}}_S( \sigma _{T_{\mathcal {\overline{E}}}}(v_j))) \}$ are incomparable in *S*. Therefore, (M2.iv) is satisfied. $\square$

As in the binary case, we obtain

#### **Theorem 5.7**

*There is a restricted reconciliation map for a gene tree* $(T;t, \sigma )$ *on* $\mathbb {G}$ *that satisfies also (O3.A) and some species tree on* $\mathbb {S}= \sigma (\mathbb {G})$ *if and only if the triple set* $\mathcal {S}(T; t,\sigma )$ *is consistent*.

### Algorithm

**Fig. 4:**
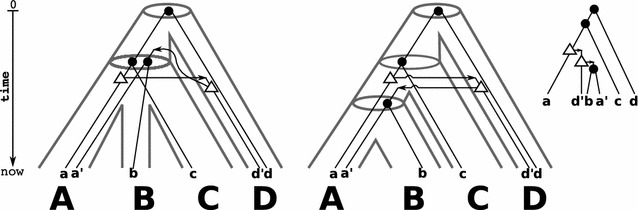
From the binary gene tree $(T;t,\sigma )$ (*right*) we obtain the species triples $\mathcal {S}(T;t,\sigma ) = \{\mathsf {(AB|D)},\mathsf {(AC|D)}\}$. Shown are two (*tube-like*) species trees (*left and middle*) that display $\mathcal {S}(T;t,\sigma )$. The respective reconciliation maps for *T* and *S* are given implicitly by drawing *T* within the species tree *S*. The left tree *S* is least resolved for $\mathcal {S}(T;t,\sigma )$. Although there is even a unique reconciliation map from *T* to *S*, this map is not time-consistent. Thus, no time-consistent reconciliation between *T* and *S* exists. On the other hand, for *T* and the middle species tree $S'$ (that is a refinement of *S*) there is a time-consistent reconciliation map. Fig. 2 provides an example that shows that also least-resolved species trees can have a time-consistent reconciliation map with gene trees

The proof of Lemmas 5.3 and 5.6 is constructive and we summarize the latter findings in Algorithm 1, see Fig. 2 for an illustrative example.

#### **Lemma 5.8**

*Algorithm 1 returns a species tree S for a binary gene tree* $(T;t,\sigma )$ *and a reconciliation map* $\mu$ *in polynomial time, if one exists and otherwise, returns that there is no species tree for* $(T;t,\sigma )$.

If $(T;t,\sigma )$ is non-binary but satisfies Condition (O3.A), then Algorithm 1 returns a species tree *S* for $(T;t,\sigma )$ and a restricted reconciliation map $\mu$ in polynomial time, if one exists and otherwise, returns that there is no species tree for $(T;t,\sigma )$.

#### *Proof*

Theorem 5.4 and the construction of $\mu$ in the proof of Lemmas 5.3 and 5.6 implies the correctness of the algorithm.

For the runtime observe that all tasks, computing $\mathcal {S}(T;t,\sigma )$, using the BUILD algorithm [64, 68] and the construction of the map $\mu$ [10, Cor.7] can be done in polynomial time. $\square$

In our examples, the species trees that display $\mathcal {S}(T; t,\sigma )$ is computed using the $\mathcal {O}(|L_R||R|)$ time algorithm BUILD, that either constructs a tree *S* that displays all triples in a given triple set *R* or recognizes that *R* is not consistent. However, any other supertree method might be conceivable, see [65] for an overview. The tree *T* returned by $\texttt {BUILD}$ is least resolved, i.e., if $T'$ is obtained from *T* by contracting an edge, then $T'$ does not display *R* anymore. However, the trees generated by $\texttt {BUILD}$ do not necessarily have the minimum number of internal vertices, i.e., the trees may resolve multifurcations in an arbitrary way that is not implied by any of the triples in *R*. Thus, depending on *R*, not all trees consistent with *R* can be obtained from $\texttt {BUILD}$. Nevertheless, in [11, Prop. 2(SI)] the following result was established.

#### **Lemma 5.9**

*Let R be a consistent triple set. If the tree T obtained with* $\texttt {BUILD}$ *applied on R is binary, then T is a unique tree on* $L_R$ *that displays R, i.e., for any tree* $T'$ *on* $L_R$ *that displays R we have* $T'\simeq T$.

**Fig. 5:**
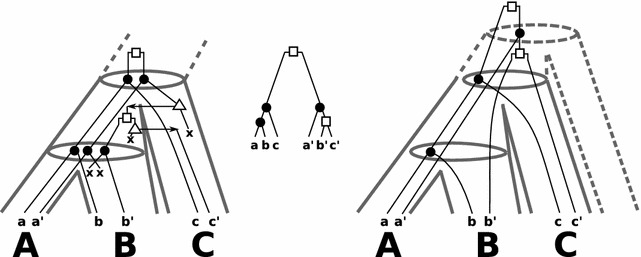
Shown is a binary and biologically feasible gene tree $(T;t,\sigma )$ (*center*) that is obtained as the observable part of the true scenario (*left*). However, there is no reconciliation map for $(T;t,\sigma )$ to any species tree according to Def. 2 because $\mathcal {S}(T;t,\sigma )$ is inconsistent. Nevertheless, a relaxed reconciliation map $\mu$ between $(T;t,\sigma )$ and the species tree exists (*right*). However, this map does not satisfy Lemma 4.2(2) since $\mu (a')=A$ and $\mu ({\text {lca}}_{T_{\mathcal {\overline{E}}}}(b',c'))$ are comparable. See text for further details


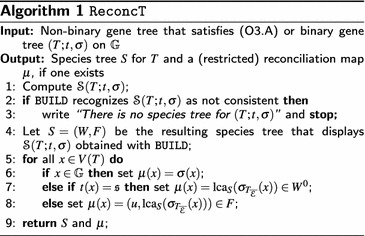


So-far, we have shown that event-labeled gene trees $(T;t,\sigma )$ for which a species tree exists can be characterized by a set of species triples $\mathcal {S}(T;t,\sigma )$ that is easily constructed from a subset of triples displayed in *T*. From a biological point of view, however, it is necessary to reconcile a gene tree with a species tree such that genes do not “travel through time”. In [63], the authors gave algorithms to check whether a given reconciliation map $\mu$ is time-consistent and for the construction of a time-consistent reconciliation maps, provided one exists. These algorithms require as input an event-labeled gene tree and species tree. Hence, a necessary condition for the existence of time-consistent reconciliation maps is given by consistency of the species triple $\mathcal {S}(T;t,\sigma )$ derived from $(T;t,\sigma )$. However, there are possibly exponentially many species trees that are consistent with $\mathcal {S}(T;t,\sigma )$ for which some of them have a time-consistent reconciliation map with *T* and some not, see Fig. 4. The question therefore arises as whether there is at least one species tree *S* with time-consistent map, and if so, construct *S*.

## Limitations of informative triples and reconciliation maps

**Fig. 6:**
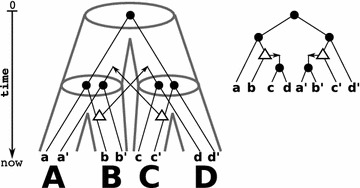
Shown is a (*tube-like*) species trees *S* with reconciled gene tree $(T;t,\sigma )$ (taken from [63]). The informative triple set $\mathcal {S}(T;t,\sigma )$ is consistent and application of Lemma 5.9 shows that *S* is unique. Moreover, the reconciliation map $\mu$ is unique, however, not time-consistent. Thus, although $\mathcal {S}(T;t,\sigma )$ is consistent, there is no *time-consistent* reconciliation map for $(T;t,\sigma )$ and *S*. Nevertheless, it can be shown that $(T;t,\sigma )$ is biologically feasible

In “Non-binary gene trees” section we have already discussed that consistency of $\mathcal {S}(T;t,\sigma )$ cannot be used to characterize whether there is a reconciliation map that doesn’t need to satisfy (M2.iv) for some non-binary gene tree, see Fig. 3. In particular, Fig. 3 shows a biologically feasible binary gene trees (center-left) for which, however, neither a reconciliation map nor a restricted reconciliation map exists.

A further simple example is given in Fig. 5. Consider the “true” history of the gene tree that evolves along the (tube-like) species tree in Fig. 5 (left). The set of extant genes $\mathbb {G}$ comprises $a,a',b,b',c$ and $c'$ and $\sigma$ maps each gene in $\mathbb {G}$ to the species (capitals below the genes) $A,B,C\in \mathbb {S}$. For the observable gene tree $(T;t,\sigma )$ in Fig. 5 (center) we observe that $\mathcal {R}_0 = \{\mathsf {(ab|c)},\mathsf {(b'c'|a')}\}$ and thus, one obtains the inconsistent species triples $\mathcal {S}(T;t,\sigma ) = \{\mathsf {(AB|C)},\mathsf {(BC|A)}\}$. Hence, Theorem 5.4 implies that there is no species tree for $(T;t,\sigma )$. Note, $(T;t,\sigma )$ satisfies also Condition (O3.A). Hence, Theorem 5.7 implies that no restricted reconciliation map to any species tree exists for $(T;t,\sigma )$. Nevertheless, $(T;t,\sigma )$ is biologically feasible as there is a true scenario that explains the gene tree.

Now consider the gene tree $(T;t,\sigma )$ in Fig. 6 (right). The set $\mathcal {S}(T;t,\sigma )$ is consistent. Both, the species trees *S* that displays all informative triples and the reconciliation map $\mu$ from $(T;t,\sigma )$ to *S*, are unique. However, $\mu$ is not time-consistent. Uniqueness of *S* and $\mu$ implies that there is no time-consistent reconciliation map for $(T;t,\sigma )$ to any species tree. Thus, consistency of $\mathcal {S}(T;t,\sigma )$ does not imply the existence of time-consistent reconciliation maps. It can be shown that $(T;t,\sigma )$ is biologically feasible.

Finally, we shortly discuss a relaxation of Condition (M2.i). Lemma 4.1 implies that $\mu (x)={\text {lca}}_S(\sigma _{T_{\mathcal {\overline{E}}}}(x))$ is the lowest possible choice for the image of a speciation vertex. Nevertheless, it is possible to relax this condition, i.e., we could allow for speciation vertices *x* that $\mu (x) \succeq _S {\text {lca}}_S(\sigma _{T_{\mathcal {\overline{E}}}}(x))$. Indeed, there are relaxed reconciliation maps for $(T;t,\sigma )$ and *S*, although no (restricted) reconciliation map for $(T;t,\sigma )$ to any species tree exists, see Fig. 5 (right). This example shows that the existence of relaxed reconciliation maps cannot be characterized by means of consistency of the informative triples in $\mathcal {S}(T;t,\sigma )$. However, it might be of interest for future research to investigate this generalization in more detail and to understand to what extent relaxed reconciliation maps imply biologically feasibility.

We summarize the latter observations:

1. Consistency of $\mathcal {S}(T;t,\sigma )$ is equivalent to the existence of a (restricted) reconciliation map. Thus, if $\mathcal {S}(T;t,\sigma )$ is consistent, then there is also a relaxed reconciliation map. The converse is not true in general.
2. Existence of time-consistent (restricted) reconciliation maps implies the existence of (restricted) reconciliation maps and thus, consistency of $\mathcal {S}(T;t,\sigma )$. The converse is not true in general.
3. If $(T;t,\sigma )$ does not contain HGT-events and $\mathcal {S}(T;t,\sigma )$ is consistent, then $(T;t,\sigma )$ is biologically feasible. The converse is not true in general.
4. If $(T;t,\sigma )$ contains HGT-events and there is a time-consistent reconciliation map for $(T;t,\sigma )$ to some species tree, then $(T;t,\sigma )$ is biologically feasible. The converse is not true in general.

## Conclusion and open problems

Event-labeled gene trees can be obtained by combining the reconstruction of gene phylogenies with methods for orthology and HGT detection. We showed that event-labeled gene trees $(T;t,\sigma )$ for which a species tree exists can be characterized by a set of species triples $\mathcal {S}(T;t,\sigma )$ that is easily constructed from a subset of triples displayed in *T*.

We have shown that biological feasibility of gene trees cannot be explained in general by reconciliation maps, that is, there are biologically feasible gene trees for which no reconciliation map to any species tree exists.

We close this contribution by stating some open problems that need to be solved in future work.

1. Are all event-labeled gene trees $(T;t,\sigma )$ biologically feasible? If not, how are biologically feasible gene trees characterized and what is the computational complexity to recognize them?
2. The results established here are based on informative triples provided by the gene trees. If it is desired to find “non-restricted” reconciliation maps (those for which Condition (M2.iv) is not required) for non-binary gene trees the following question needs to be answered: How much information of a non-restricted reconciliation map and a species tree is already contained in *non-binary* event-labeled gene trees $(T;t,\sigma )$? The latter might also be generalized by considering relaxed reconciliation maps (those for which $\mu (x)\succ _S {\text {lca}}_S(\sigma _{T_{\mathcal {\overline{E}}}}(x))$ for speciation vertices *x* or any other relaxation is allowed).
3. Our results depend on three axioms (O1)–(O3) on the event-labeled gene trees that are motivated by the fact that event-labels can be assigned to internal vertices of gene trees only if there is observable information on the event. The question which event-labeled gene trees are actually observable given an arbitrary, true evolutionary scenario deserves further investigation in future work, since a formal theory of observability is still missing.
4. The definition of reconciliation maps is by no means consistent in the literature. For the results established here we considered three types of reconciliation maps, that is, the “usual” map as in Def. 2 (as used in, e.g. [10, 31, 54, 63]), a restricted version (as used in, e.g. [12, 48]) and a relaxed version. However, a unified framework for reconciliation maps is desirable and might be linked with a formal theory of observability.
5. “Satisfiable” event-relations $R_1,\dots ,R_k$ are those for which there is a representing gene tree $(T;t,\sigma )$ such that $(x,y)\in R_i$ if and only if $t({\text {lca}}(x,y))=i$. They are equivalent to so-called unp two-structures [6]. In particular, if event-relations consist of orthologs, paralogs and xenologs only, then satisfiable event-relations are equivalent to directed cographs [6]. Satisfiable event-relations $R_1,\dots ,R_k$ are “S-consistent” if there is a species tree *S* for the representing gene tree $(T;t,\sigma )$ [12, 48]. However, given the unavoidable noise in the input data and possible uncertainty about the true relationship between two genes, one might ask to what extent the work of Lafond et al. [12, 48] can be generalized to determine whether given “partial” event-relations are S-consistent or not. It is assumable that subsets of the informative species triples $\mathcal {S}(T;t,\sigma )$ that might be directly computed from such event-relations can offer an avenue to the latter problem. Characterization and complexity results for “partial” event-relations to be satisfiable have been addressed in [74].
6. In order to determine whether there is a time-consistent reconciliation map for some given event-labeled gene tree and species trees fast algorithms have been developed [63]. However, these algorithms require as input a gene tree $(T;t,\sigma )$ *and* a species tree *S*. A necessary condition to a have time-consistent (restricted) reconciliation map to some species tree is given by the consistency of the species triples $\mathcal {S}(T;t,\sigma )$. However, in general there might be exponentially many species trees that display $\mathcal {S}(T;t,\sigma )$ for which some of them may have a time-consistent reconciliation map with $(T;t,\sigma )$ and some might have not (see Fig. 4 or [63]). Therefore, additional constraints to determine whether there is at least one species tree *S* with time-consistent map, and if so, construct *S*, must be established.
7. A further key problem is the reliable identification of horizontal transfer *events*. In principle, likely genes that have been introduced into a genome by HGT can be identified directly from sequence data [75]. Sequence composition often identifies a gene as a recent addition to a genome. In the absence of horizontal transfer, the similarities of pairs of true orthologs in the species pairs (A,B) and (A,C) are expected to be linearly correlated. Outliers are likely candidates for HGT events and thus can be “relabeled”. However, a more detailed analysis of the relational properties of horizontally transferred genes is needed.
